# Spontaneous Tumor Regression and Reversion: Insights and Associations with Reduced Dietary Phosphate

**DOI:** 10.3390/cancers16112126

**Published:** 2024-06-03

**Authors:** Ronald B. Brown

**Affiliations:** School of Public Health Sciences, University of Waterloo, Waterloo, ON N2L 3G1, Canada; r26brown@uwaterloo.ca

**Keywords:** spontaneous tumor regression, tumor reversion, dietary phosphate overload, kinases and phosphatases, dysregulated phosphate metabolism, tumor microenvironment, tumor encapsulation, fasting-mimicking diet, sickness-associated anorexia, autophagy

## Abstract

**Simple Summary:**

In spontaneous tumor regression, tumors shrink and disappear without conventional treatments. This phenomenon challenges the view that cancer is an irreversible genetic disease and that the only treatment option is to kill cancer cells or surgically remove them. In tumor reversion, cancer cells have been shown to return to normal cells when they are transplanted into a normal cellular environment. Additionally, people consuming a Western diet ingest excessive amounts of dietary phosphate, and a dysregulated oversupply of phosphate can be transported into cells, stimulating the cellular growth that forms tumors. Based on reviewed evidence, this paper proposes that reducing excessive dietary phosphate potentially activates tumor regression and reversion, as components of cancer cells are self-digested. Furthermore, fevers and fasting-mimicking diets are associated with tumor regression, which also may be initiated by reduced phosphate intake. Studies are needed to test dietary phosphate reduction in tumor regression and reversion to improve cancer patient survival.

**Abstract:**

Tumors that spontaneously shrink from unknown causes in tumor regression, and that return to normal cells in tumor reversion, are phenomena with the potential to contribute new knowledge and novel therapies for cancer patient survival. Tumorigenesis is associated with dysregulated phosphate metabolism and an increased transport of phosphate into tumor cells, potentially mediated by phosphate overload from excessive dietary phosphate intake, a significant problem in Western societies. This paper proposes that reduced dietary phosphate overload and reregulated phosphate metabolism may reverse an imbalance of kinases and phosphatases in cell signaling and cellular proliferation, thereby activating autophagy in tumor regression and reversion. Dietary phosphate can also be reduced by sickness-associated anorexia, fasting-mimicking diets, and other diets low in phosphate, all of which have been associated with tumor regression. Tumor reversion has also been demonstrated by transplanting cancer cells into a healthy microenvironment, plausibly associated with normal cellular phosphate concentrations. Evidence also suggests that the sequestration and containment of excessive phosphate within encapsulated tumors is protective in cancer patients, preventing the release of potentially lethal amounts of phosphate into the general circulation. Reducing dietary phosphate overload has the potential to provide a novel, safe, and effective reversion therapy for cancer patients, and further research is warranted.

## 1. Introduction

A handful of recently published papers on the spontaneous regression and reversion of tumors has revived interest in unexplained tumor disappearance, a historical phenomenon observed over many centuries in plants, animals, and humans [1,2,3,4,5,6,7,8]. The spontaneous regression of cancer in humans was defined in 1956 by Dr. Warren H. Cole and Dr. Tilden C. Everson as “the partial or complete disappearance of a malignant tumor in the absence of all treatment, or in the presence of therapy which is considered inadequate to exert a significant influence on neoplastic disease” [9]. Uncovering the elusive cause of spontaneous tumor regression is tantamount to solving a biomedical whodunit. Even more mysteriously, tumor reversion has appeared to magically transform cancer cells to normal cells under experimental conditions [1]. Irrefutable evidence of tumor regression and reversion challenges the somatic mutation theory, which proposes that cancer is an irreversible genetic disease [1]. The somatic mutation theory is based on the assumption that “a somatic cell in the adult organism would undergo successive DNA mutations, and these mutations would be responsible for the cancer phenotype” [10]. Yet, only 30–40% of cancer cells contain genetic mutations, and similar mutations occur in normal cells without cancer [2].

The assumed irreversibility of cancer has guided the research and development of treatments that destroy cancer cells in patients, including chemotherapy, immunotherapy, radiotherapy, and surgery. Yet, current treatments do not remove the underlying cause of cancer. “Cancer therapy is still based on the millenary paradigm that establishes that in order to achieve a cure, the complete eradication of cancer cells must be achieved”, but critics have called for the development of cancer treatments that incorporate a more holistic approach toward patients’ needs [11]. “Knowledge of how a tumor heals itself would be immensely helpful in developing more effective therapeutic modalities” [4]. Additionally, studying the causes of the spontaneous regression and reversion of tumors could contribute to a paradigm shift in cancer therapy and improve the survival and welfare of cancer patients.

Rising cancer incidence in people under 50 years of age is of immediate concern [12], and new approaches are needed to reverse this alarming trend, including a greater focus on nutrition and diet as advised by the World Cancer Research Fund/American Institute for Cancer Research [13]. A healthful diet supplies the body with building materials and fuel for energy, but nutrients and related metabolites also help manage many cellular processes in the body, including protein modification, cell signaling, and gene expression [14]. The dysregulation of these cellular processes leads to dysregulated cellular function in diseases like cancer, implying the involvement of dietary nutrients. For example, chronic nutrient overload can burden cell organelles with an excessive supply of metabolites that overactivate signaling pathways for energy storage and tissue biosynthesis [15]. These types of findings suggest that cancer is a metabolic disease caused by dysregulated metabolic functions [16].

The evidence reviewed in the present paper investigates the associations of spontaneous regression and reversion of tumors with the dietary mineral phosphorus, an essential nutrient which is acquired from the diet in the form of phosphate (PO_4_^3−^) [17]. A grounded theory literature review method [18] was used to search concepts in Google, Google Scholar, Pub Med, and Scopus, related to diet, cancer, and the spontaneous regression and reversion of tumors, including concepts involving dietary phosphate overload, dysregulated phosphate metabolism in tumorigenesis, and cellular autophagy. All data from credible sources are included in this grounded theory study regardless of the type and date of the data. A comparative analysis of reviewed findings was used to inductively group concepts into categories and themes, which were iteratively synthesized into an explanatory grounded theory of tumor regression and reversion. The paper’s narrative review presents new knowledge and novel insights and proposes that the spontaneous regression and reversion of tumors is potentially associated with reduced dietary phosphate and reregulated phosphate metabolism.

Importantly, the article does not suggest that patients should replace conventional therapies for cancer with a low-phosphate diet for tumor regression/reversion. Clinical evidence demonstrating a causative effect of reduced dietary phosphate on tumor regression is currently lacking, and other factors may be involved. The aim of the review is to synthesize fragmentary pieces of available evidence from the literature into a coherent rationale for conducting further preclinical research and testing. The long-term objective proposed by the paper is to increase options for adjuvant therapies available to cancer patients based on reliable clinical trial evidence. This paper intends to provide an important foundational starting point towards those goals.

## 2. Tumor Regression

In their 1956 paper, doctors Cole and Everson clarified that spontaneous regression need not imply complete tumor disappearance, “nor that spontaneous regression is synonymous with cure” [9]. Furthermore, the authors mentioned cases in which a spontaneous regression of a tumor in one area did not affect tumors that “flourished unchecked in other areas of the body or reappeared at a later time”, implying the potential involvement of a systemic causative mechanism. Doctors Cole and Everson also proposed factors that are plausibly responsible for the spontaneous regression of tumors, including acute infection and fever (immune responses), endocrine factors, removal of a “carcinogenic agent”, and “interference of the nutrition of the tumor”.

After serving as President of the American Cancer Society in 1959 [19], Dr. Cole wrote in the Annals of the New York Academy of Sciences in 1974 that the use of the word “spontaneous” in cancer regression is a misnomer [20]. He explained that such cases of cancer regression have an unknown cause, and therefore “idiopathic” cancer regression is a more appropriate term to describe this phenomenon. Dr. Cole also pointed out the difficulty of accurately estimating the incidence rate of spontaneous cancer regression, which he suggested is far more frequent than 1 out of 80,000 to 100,000 cancer patients. Spontaneous regression has most often been documented for lymphomas, neuroblastomas, melanomas, renal cell carcinomas, and testicular malignancies, and less often for breast cancer and lung cancer [3]. However, Papac noted the reason that the spontaneous regression of breast cancer is rarely reported is because it is so commonly treated [21]. The spontaneous regression of gastrointestinal malignancies was also more than twice as common in men compared to women in a recent review of 390 cases [22].

Dr. Cole hypothesized that “regression occurs when the factors that cause the growth are no longer present” [20]. In further discussing the elimination of a carcinogen as a cancer-regression factor, Dr. Cole presented evidence in which bladder cancer regression was induced when patients’ ureters were detached from the bladder and transplanted to the colon. He wrote, “there appears to have been something in the urine either responsible for the development of, or the continuing growth of, the bladder cancer”,—i.e., a carcinogenic substance eliminated by the kidneys. The present paper proposes that a potential growth-promoting factor fitting Dr. Cole’s prescient hypothesis is the renal excretion of dietary phosphate, which is a metabolite associated with tumorigenesis when consumed in excessive amounts—a factor also known as dietary phosphate overload.

### 2.1. Dietary Phosphate Overload

Phosphate overload in the human body is defined as “a state where the phosphorus load is higher than is physiologically necessary”, and causes of phosphate overload include a diet that is high in phosphorus and/or insufficient renal function to regulate phosphate serum levels [23]. Serum phosphate is regulated by an endocrine system consisting of the kidneys, bones, parathyroid glands, and intestines, which is coordinated by endocrine hormones: fibroblast growth factor 23 (FGF23), klotho, parathyroid hormone (PTH), and 1,25-dihydroxyvitamin D3. Excess phosphate is excreted in the urine and is also temporarily stored in a reservoir “pool” within the body, mostly as hydroxyapatite (calcium phosphate) in long bones and in extracellular fluid as inorganic phosphate (Pi) [24]. When intracellular and extracellular levels of phosphate rise too high, a condition known as phosphate toxicity can negatively affect most of the major organ systems of the body [25].

Dietary phosphate overload from excessive phosphate intake is increasingly associated with chronic diseases including cancer. Importantly, chronic kidney disease (CKD) is associated with increased cancer risk [26]. A murine model of CKD showed that dietary phosphate overload induced inflammation, vascular calcification, malnutrition, and premature death [27]. Researchers advise that “dietary strategies are required to reduce dietary phosphate overload and improve human health” [28]. The natural whole foods that are highest in phosphorus are meats, poultry, seafood, dairy, cheese, eggs, nuts and seeds, whole grains, and legumes [29]. Table A2 in Appendix B lists the food items highest in phosphorus. Phosphate additives are also prevalent in processed foods including baked goods, colas, processed meats, and fast foods. Refined lipids, on the other hand, have most minerals removed, as do refined carbohydrates. Among whole unrefined foods, fresh fruits and vegetables are generally lowest in phosphate. A list of foods lowest in phosphorus is included in Table A3 in Appendix C. Dietary reference intakes for the phosphorus needed by the population are listed in Table A1 in Appendix A. Surveys show that the average adult consumes approximately double the 700 mg of phosphorus per day recommended by the Institute of Medicine [30].

A 2020 systematic review and meta-analysis investigated fruit and vegetable consumption associated with prognosis of cancer survivors who are at increased risk for a second primary tumor [31]. The researchers found that fruit and vegetable intake was associated with reduced mortality in patients with ovarian cancer, and vegetable intake was also associated with reduced mortality in patients with head and neck cancers. Additionally, a study of the BioBank Japan Project found that low intake of green leafy vegetables was associated with higher mortality in male patients with colorectal cancer [32]. Overall, observational studies show that reduced cancer risk is associated with plant-based diets that are higher in fruits and vegetables and lower in animal-based foods [33]. These findings generally imply that fruits and vegetables, in addition to providing important nutrients, tend to lower the overall intake of dietary phosphate.

### 2.2. Immunotherapy, Fevers, and Fasting

In 1976, Dr. Warren H. Cole delivered an address at the John Hopkins Medical Institutions on the importance of finding the cause for the spontaneous regression of cancer [34]. He stated, “If we are going to make progress in finding answers, I suspect we will have to reach outside the realm of accepted facts about cancer”.

#### 2.2.1. Immunotherapy

In his final article on efforts to explain the spontaneous regression of cancer in 1981, Dr. Cole wrote that he was “convinced stimulation of the immune process is the most important factor”, and he was enthusiastic about interferon therapy for cancer [35]. Nevertheless, Dr. Cole also reiterated that “elimination of carcinogens appears important”, based on the findings of bladder cancer regression associated with the diversion of a potential carcinogen in urine. Since then, immunotherapies have continued to face challenges to meet expectations for cancer treatment efficacy [36,37], and tumors’ evasion of attacks from the immune system is now well recognized [38]. These research findings highlight the need for investigators to refocus on the elimination of carcinogens in the spontaneous regression of cancer, including eliminating dietary factors like phosphate overload.

Fortunately, Dr. Cole’s comments on bladder cancer have since been corroborated by additional evidence involving phosphorus. For example, the 2011 Belgian case–control study of bladder cancer found that the highest intake of dietary phosphate was associated with a statistically significant two-fold increased odds of bladder cancer in older adults [39]. More recently, phosphate in urine from patients with bladder cancer was associated with greater toxicity and altered cell proliferation [40]. As previously mentioned, phosphate is dysregulated in CKD, and a higher risk of bladder cancer is associated with CKD in non-dialysis patients [41].

#### 2.2.2. Fevers

In their review of the spontaneous regression of cancer, Radha and Lopus mentioned that tumor regression following fevers and acute infections were reported by the ancient Egyptians [4]. The authors also described how, in 1891, Dr. William Bradley Coley induced fevers in cancer patients using injections of bacteria, but that subsequent tumor regression was inconsistent, and the treatment had adverse effects. Similar responses to infection have been suggested to account for spontaneous tumor regression following a systemic reaction to the COVID-19 vaccination with “intense reactogenicity” [42]. Although these cases of tumor regression are associated with the stimulation of the immune system, other related factors should be considered. For example, loss of appetite and reduced dietary intake during acute infections and febrile conditions, including reduced intake of dietary phosphate, should be investigated.

Importantly, appetite suppression or sickness-associated anorexia in infected humans and other species is part of an immune response that defends the survival of the infected host—a nutritional immunology factor [43]. Studies have shown that feeding during sickness could aggravate the illness and that underfeeding can be beneficial. Sickness-associated anorexia also promotes autophagy. Derived from the Greek words for “self” and “eat”, autophagy is defined by Ebrahimi et al. as a “process by which eukaryotic cells degrade large cellular components into substrates that subsequently can be either used as fuel source, or utilized for the synthesis of critical cellular components” [44]. The researchers also found that dietary phosphate restriction activates autophagy in yeast, implying that the association of the spontaneous regression of tumors with autophagy and sickness-associated anorexia is potentially mediated by dietary phosphate restriction.

In addition to associations with the regression of solid tumors, fever was associated with spontaneous remission in over 90% of acute myeloid leukemia cases in a 2014 review [45]. The spontaneous remission of acute myelomonocytic leukemia includes lowered blood neutrophil and platelet counts without chemotherapy, and remission was recently documented in a case study of a woman with fever and a severe COVID-19 infection [46]. However, the authors noted that spontaneous remission usually does not endure in cases of acute myeloid leukemia, and remission lasted only six months in their patient.

#### 2.2.3. Fasting

Cancer cells appear to be more vulnerable to nutrient deprivation than normal cells [47], plausibly related to a weaker blood supply to cancer cells due to the highly abnormal structure and function of the tumor vasculature [48]. Consequently, nutrient deprivation through fasting can reduce levels of growth factors and metabolites and create “environments that can reduce the capability of cancer cells to adapt and survive” [47]. Additionally, autophagy “plays a critical role in regulating cellular nutrient status in a fasted state” [44], and fasting-mimicking diets are associated with tumor regression [49]. Fasting-mimicking diets have also improved clinical outcomes in cancer patients in combination with conventional antineoplastic treatments [50,51].

Originally developed in 2015 by Brandhorst et al., a fasting-mimicking diet was shown to reduce cancer incident in C57BL/6 mice, and the diet lowered cancer risk factors and biomarkers in humans [52]. In the pilot clinical study of the diet, participants were randomized to receive a plant-based low-protein fasting-mimicking diet for five days a month and a total of three months. The diet consisted of approximately 1090 calories consumed on the first day and 725 calories consumed on each of the next four days. The researchers designed the diet “to provide 34–54% of the normal caloric intake with a composition of at least 9–10% proteins, 34–47% carbohydrates, and 44–56% fat”. The participants consumed their usual diet during the rest of the study.

Future research of dietary phosphate restriction associated with autophagy and fasting therapy for cancer regression is warranted, including investigations of effects from nutrients other than phosphorus. Additionally, appetite loss is a common side effect of cancer treatments [53], and research should investigate sickness-associated anorexia and autophagy as confounding factors associated with reduced dietary phosphate in tumor regression during cancer treatments.

## 3. Autophagy and Phosphate in Tumor Regression and Reversion

The National Cancer Institute noted that autophagy may protect cancer cells “by providing extra nutrients to them or by keeping anticancer drugs or other substances from destroying them”, but no mention was made of autophagy in tumor regression and reversion [54]. Gluconeogenesis keeps cancer cells supplied with glucose, which is derived from lactate and amino acids [55], and autophagy is a necessary mechanism in gluconeogenesis for maintenance of tumors [56]. In addition to promoting tumor growth in the later stages of cancer, autophagy plays a role in tumor prevention by suppressing cancer initiation in the early stages of tumorigenesis [57]. Although these autophagy mechanisms affect tumor growth factors, they do not fully explain autophagy in idiopathic tumor regression. Hypothetically, cancer cells may degrade intracellular components by applying the same basic principles of autophagy as other cells.

In a review of the basic principles of autophagy by Kundu and Thompson, the authors mentioned the role of lysosomes in autophagy as part of the “process by which cellular components are sequestered within the vesicular system and delivered to lysosomes for degradation and recycling of bioenergetic components” [58]. An important distinction between autophagy and autolysis is that enzymes in lysosomes are used to degrade intracellular components in autophagy, whereas the same enzymes are used to destroy the entire cell in autolysis [59]. Evidence of cancer cell reversion in the present paper shows that the cell is not destroyed, suggesting that autophagy rather than autolysis is the main mechanism involved in tumor reversion.

Kundu and Thompson described the steps of autophagy, beginning with the selection and packaging of cargo for degradation, with the subsequent exportation and recycling of “metabolic building blocks” [58]. For example, bioenergetic and biosynthetic phosphate substrates are recycled through autophagy for incorporation into adenosine triphosphate (ATP) and nucleotides, which support the cell survival from energy starvation and depleted nucleotide pools [60]. Kundu and Thompson also noted that the type of stimulus determines if autophagy targets nonspecific or specific contents of the cytoplasm for degradation. The authors added that autophagy can be an “adaptive response” to a stimulus, and that a change in the availability of nutrients signals the initiation of nonselective autophagy. Additionally, the authors listed cellular stresses that dramatically induce autophagy, including “growth factor withdrawal”. These principles of autophagy appear consistent with the withdrawal of phosphate overload in tumor regression and reversion.

In addition to recycling substrates, an additional function of autophagy is the elimination of intracellular macromolecules [61]. A mechanism, by which autophagy targets specific contents in the cytoplasm of cancer cells, involves acid phosphatases contained in lysosomes which break apart (lyse) macromolecules during autophagy [62]. Intracellular phosphate is stored as macromolecules of inorganic polyphosphate in some types of cancer cells [63], and phosphatase has been shown to catalyze the breakdown or hydrolysis of polyphosphate [64]. Studies should investigate the potential suppression of acid phosphatase in lysosomes as polyphosphate accumulates in cancer cells during tumorigenesis, as well as the activation of acid phosphatase during autophagy that breaks apart polyphosphate stored in cancer cells. Summing up, the withdrawal of phosphate overload is proposed to stimulate autophagy in cancer cells, including the specific hydrolysis of polyphosphate macromolecules. Also, bioenergetic and biosynthetic phosphate substrates may be eliminated or recycled.

## 4. Experimental Evidence of Tumor Reversion

In 1959, Pierce and Dixon used a mouse model of testicular teratocarcinoma to restore a normal phenotype in malignant embryonic cells by modifying the cell microenvironment [65]. In 1975, Mintz and Illmensee demonstrated that malignant embryonic cells in a mouse model produced normal functioning cells when transplanted into a normal environment [66]. Mintz implied that tumorigenesis was due to the cell environment, not to autonomous changes in the cell itself [67]. Other research confirmed that an embryonic microenvironment has anti-tumor effects on malignant cells [68], plausibly related to the tighter regulation of biochemical factors conducive to normal growth in an embryonic microenvironment compared to dysregulated growth in a tumor microenvironment.

Notably, a “normal” cellular microenvironment in tumor reversion implies normal concentrations of phosphate. Generally, phosphate levels within mammals have been shown to range from 0.5 to 5 mM, but higher concentrations of Pi, up to 10 mM, can stimulate cell proliferation [69]. Studies are needed to investigate phosphate concentrations in the cellular microenvironment associated with facilitation of tumor reversion.

In their 2023 literature review, Pensotti, Bertolaso, and Bizzarri summarized conclusions from experimental evidence supporting tumor reversion [2], shown in Table 1.

## 5. Dysregulated Phosphate Metabolism and Tumorigenesis

The research literature on cancer describes the cancer cell’s demand for nutrients to support autonomous growth [70], yet evidence associates tumor cell growth with adaptation to cellular nutrient overload—a reflexive response dependent on an externally imposed oversupply in contrast to an independent internal demand. As a potential carcinogenic and epigenetic factor, the toxic effects of nutrient overload are well recognized in nutritional epidemiology, and cancer is also strongly associated with diet [71]. “Metabolic reprogramming of cancer cells may result in strong dependencies on nutrients that could be exploited for therapy”, and dietary interventions restricting nutrients required for tumor growth have been proposed for cancer therapy [72].

In a review of dietary phosphate consumption and tumorigenesis, Arnst and Beck Jr. noted that changes in intracellular and extracellular levels of Pi affect glucose metabolism, inflammation, and oxidative stress, which are associated with tumorigenesis and cancer progression [73]. Studies cited by the authors showed that tumors in mice and humans had greater Pi uptake and retention compared to healthy tissue. The authors also reviewed evidence showing that Pi activates cell-signaling pathways for cellular growth, and the authors noted “the possibility that Pi consumption can be manipulated to control cell growth, particularly in rapidly dividing cells”.

Importantly, Arnst and Beck Jr. emphasized “the paradox of how an element critical to essential cellular functions can, when available in excess, influence and promote a cancer phenotype”. Furthermore, the stealth ability of phosphate to hide in plain sight as an essential nutrient for cell growth, and then surreptitiously turn into a carcinogenic agent during dietary phosphate overload could explain how the association of phosphate with tumorigenesis has evaded detection by most cancer researchers. Moreover, this mechanism is potentially related to tumor regression and reversion under circumstances of reduced dietary phosphate. Although Arnst and Beck Jr. did not review regression and reversion of tumors, they suggested that dietary phosphate restriction has potential as a novel therapeutic approach to control tumorigenesis [73].

Sullivan and Vander Heiden wrote that “proliferation requires that cells accumulate sufficient biomass to grow and divide. Cancer cells within tumors must acquire a variety of nutrients, and tumor growth slows or stops if necessary metabolites are not obtained in sufficient quantities” [74]. Befittingly, the dietary mineral phosphorus is a primary nutrient for the growth of human cellular biomass [75]. As a growth factor, phosphate nutrient overload is a potential carcinogenic agent for cancer-cell proliferation within the tumor microenvironment, and a target for phosphate reduction in tumor regression and reversion.

### 5.1. Nutrient Toxicity

Excessive amounts of nutrients can become toxic, according to a 1981 article in *Nutrition Reviews* by Campbell, Allison, and Fisher [76]. The authors explained that the intake of a nutrient on a dose–response curve spans across ranges indicating deficiency, adequacy, and toxicity. They further explained that the original objective of developing guidelines for the recommended nutrient intake was to reduce the risk of deficiency diseases, but some nutrients may be consumed in amounts much greater than recommended. “The public appears to understand the possibility of adverse effects resulting from excessive consumption of calories, salt, protein, refined sugar, fat and cholesterol, and alcohol: but this concept is not extended as easily to vitamins and essential minerals”, including dietary phosphorus.

Compared to toxicity from a single megadose of nutrients, there is “greater public health concern about chronic nutrient toxicity where the effects develop more subtly and slowly” [76]. For example, Table A1 in Appendix A shows that the estimated average requirement (EAR) for phosphorus is 580 mg/day for adults, which increases to a recommended dietary allowance (RDA) of 700 mg/day to meet the needs of 97.5% of the adult population [77]. The tolerable upper intake level (UL) for phosphorus in most adults without causing harmful health effects is 4000 mg. Yet, although mean phosphorus intake within the United States adult population is well below the UL considered toxic [30], an intake of more than twice the RDA is nevertheless sufficient to associate cancer with chronic nutrient toxicity from excessive dietary phosphate intake. For instance, middle-aged women who consumed >1800 mg phosphorus were recently found to have over twice the relative risk of breast cancer incidence compared to women consuming 800–1000 mg phosphorus [78].

In their review on dietary phosphate and cancer [73], Arnst and Beck Jr. included contradictory findings of increased cancer risk in a study of mice consuming a low-phosphate diet [79]. This contradiction prompted Arnst and Beck Jr. to speculate that the association of dietary phosphate levels and cancer risk could follow a U-shaped curve. However, the cited mouse study did not appear to control for the high amount of phosphate in casein fed to the mice, as advised by the American Institute of Nutrition’s (AIN) final report of the AIN-93 rodent diet [80]. Consequently, equal amounts of casein (200 g/kg diet) were inadvertently fed to both the normal and low-phosphate groups of the study. In comparison with casein, fat in cow’s milk contains no phosphorus, which could contribute to the “anticarcinogenic” effects of the fat components of cow’s milk in animal experiments [81]. Interestingly, neuroblastoma has a high propensity for spontaneous regression and is much more frequent in infants [82,83], perhaps related to the variable intake of cow’s milk which is high in phosphorus—by comparison, breast milk is approximately six times lower in phosphorus [84]. More studies are needed to investigate changes in dietary phosphate overload and the spontaneous regression of neuroblastoma in infants.

### 5.2. Phosphate in the Tumor Microenvironment

During tumorigenesis, excess phosphate in the tumor microenvironment upregulates the expression of sodium-dependent Pi transporters, NaPi2b, which sequester phosphate into cancer cells [85]. High extracellular levels of Pi in breast cancer cells (MDA-MB-231 cell line) were shown to stimulate H+-independent Pi transporters with five-fold more Pi transport than NaPi2b transporters [86].

**Figure 1:**
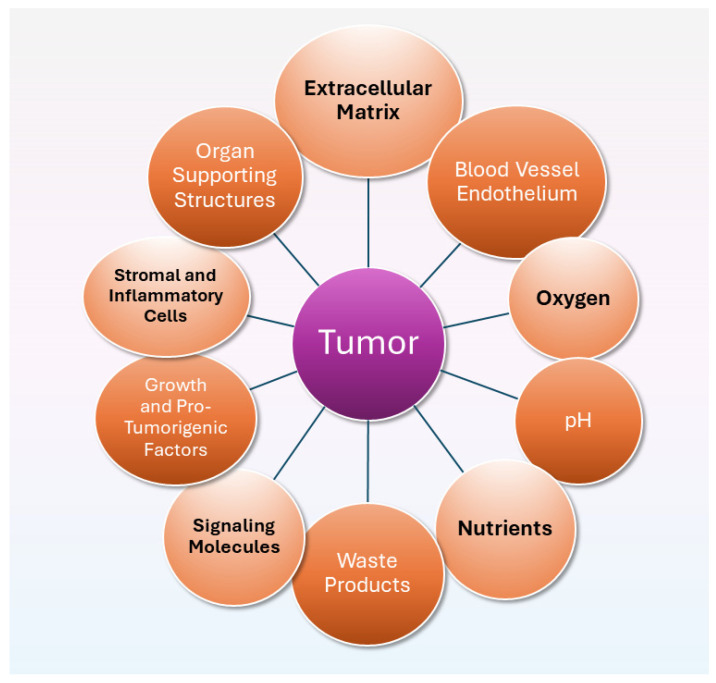
Schematic drawing of heterogeneous components within the tumor microenvironment, based on Bobko et al. [87].

In 2017, West Virginia University researchers used an in vivo electron paramagnetic resonance technique in mouse models of cancer to investigate components of the tumor microenvironment, which included the extracellular matrix, blood vessel endothelium, and other heterogeneous components shown in Figure 1 [87]. The researchers found that higher levels of interstitial Pi within the tumor microenvironment were associated with metastasizing tumors. This important finding implies that metastasis is potentially a systemic disorder associated with dysregulated phosphate metabolism—providing an alternative to the seed and soil theory that proposes that metastasis spreads to secondary sites as cancer cells escape from a tumor [88]. More recently in 2023, the West Virginia University researchers used the same in vivo technique in a mouse breast-cancer model to investigate cancer biomarkers within the tumor microenvironment, including oxygen concentration and extracellular pH [89]. The researchers found that only interstitial Pi, a biomarker of deviated metabolic status, was consistently elevated during all stages of tumorigenesis up to and including malignancy.

Additionally, in 2021, South American researchers used micro X-ray fluorescence to measure phosphorus in samples of tumor tissue from the mammary glands of BALB/c mice, and the researchers found a strong correlation of phosphorus levels with active adenocarcinoma cells [90]. Other researchers exposed cancer cell samples to extracellular Pi and found that concentrations above 16 mM caused cancer cell death by apoptosis [69].

### 5.3. Kinases and Phosphatases

The phosphorylation of a protein by kinase enzymes is a post-translational modification that binds a negatively charged phosphate group to the protein’s amino acids (serine/threonine, and tyrosine) [91]. This reaction modifies the protein’s polarity and changes its configuration as it interacts with other biomolecules in cell signaling. Importantly, activation of this cell-signaling mechanism is reversible through the dephosphorylation by phosphatase enzymes. Furthermore, “the uncontrolled activation of kinases and the suppression of phosphatases has been frequently observed in cancer” [92].

**Figure 2:**
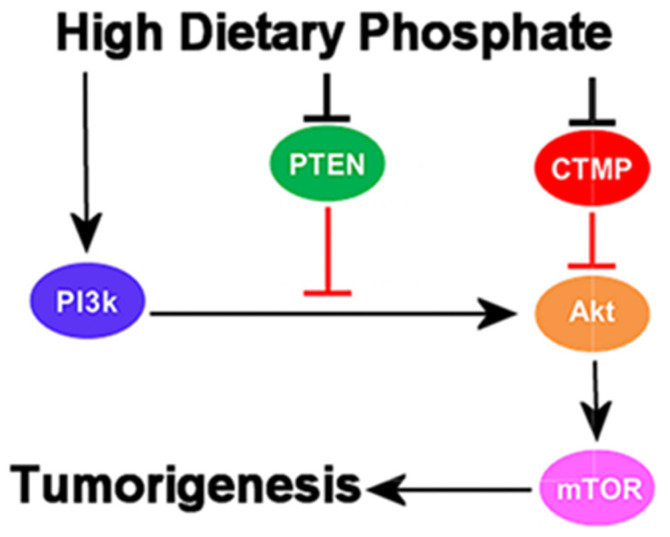
High dietary phosphate and cancer-cell signaling [96]. This flowchart shows that high dietary phosphate activates the phosphoinositide 3-kinase (PI3K)/Akt/mTOR signaling pathway while deactivating tumor suppression by PTEN and CTMP, increasing cell growth in tumorigenesis.

Over 1000 variations have been found in protein kinases in human tumors [91]. Yet, clinical trials of kinase inhibitors have been mostly unsuccessful in treating solid tumors, and some researchers suggest the necessity of simultaneously activating phosphatases such as Protein Phosphatase 2A (PP2A) [93]. However, high dietary phosphate in a mouse model of lung cancer was sufficient to inhibit expression of the tumor suppressors phosphatase and tensin homolog (PTEN) and carboxyl-terminal modulator protein (CTMP), while also activating the Akt signaling pathway (protein kinase B) [94]. These findings, illustrated in Figure 2, imply that the problem with treating cancer patients with kinase inhibitors and phosphatase activators may lie in the failure to control dietary phosphate intake, which regulates the balance of kinases and phosphatases. Moreover, experiments have shown that intestinal alkaline phosphatase activity and the expression of alkaline phosphatase 3 protein levels in the intestines of mice were increased with a low-phosphate diet compared to higher phosphate intake [95]. Based on the preceding findings, this paper proposes reducing dietary phosphate overload and reregulating phosphate metabolism to reverse uncontrolled kinase activation and phosphatase inhibition in cancer-cell signaling, thereby inducing tumor regression and reversion.

### 5.4. Nutrient Metabolism in Cancer

Although scientists have experimentally demonstrated tumor reversion by transplanting malignant cells into healthy cellular microenvironments, an understanding of the causative mechanisms of tumor reversion and regression remains elusive. Altea-Manzano et al. summarized several factors that influence nutrient metabolism in cancer [97]. These factors apply to dysregulated phosphate metabolism in tumorigenesis with potential applications to tumor regression and reversion (Table 2).

### 5.5. Tumor Metabolic-Reversal Model

**Figure 3:**
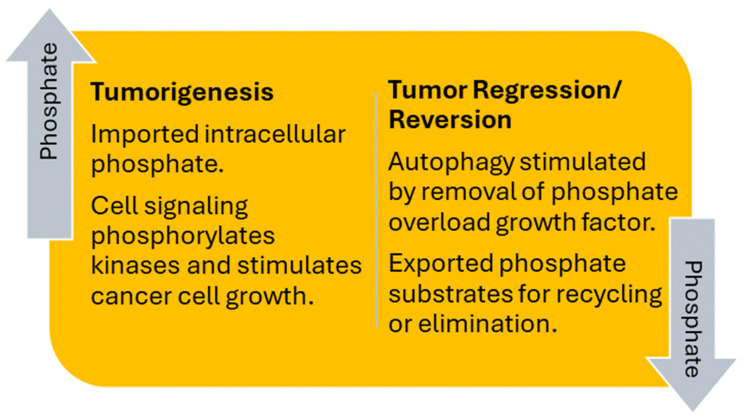
Tumor Metabolic-Reversal Model. The model shows that tumorigenesis from imported intracellular phosphate and cell signaling is reversed by removal of phosphate overload, which stimulates autophagy and recycles or eliminates phosphate substrates.

The following tumor metabolic-reversal model is based on a biological framework for cancer research developed by Schipper et al., which posits that cancer is a metabolic disease and may be reversable [98]. The model, shown in Figure 3, incorporates the metabolic principles of tumorigenesis and tumor regression/reversion, which are stimulated by high and low levels of phosphate overload, respectively. In tumorigenesis, imported intracellular phosphate increases cell signaling by phosphorylating kinases, which stimulates tumor cell growth. In tumor regression/reversion, autophagy is stimulated by the removal of the tumor growth factor when phosphate overload is reduced. Phosphate substrates from autophagy are subsequently exported from the cell and are either eliminated or recycled in the body for the bioenergetic and biosynthetic formation of new compounds (e.g., ATP and nucleotides).

## 6. Tumor Encapsulation and Phosphate Toxicity

Tumors that are encapsulated with a surrounding band of connective tissue are considered benign tumors, although not all benign tumors are encapsulated [99]. By contrast, a malignant tumor continues to grow without a well-formed capsule [100]. The foreign body hypothesis proposes that “capsule formation represents an attempt by the host to contain the tumour” in a fashion analogous to “encapsulation of a foreign body” [99]. Encapsulated tumors are associated with a protective effect in cancer patients. For example, 72 cases of encapsulated hepatocellular carcinoma resulted in “significantly better disease-free and actuarial survival times” compared to nonencapsulated cases [101]. The researchers noted that “lower extensive tumor invasiveness” was a significant factor contributing to better prognosis in encapsulated cases, and similar results were found in women with encapsulated hepatocellular carcinoma [102]. Another study using magnetic resonance imaging found that complete tumor encapsulation in patients with a large solitary hepatocellular carcinoma was a “predictor for favorable biology” [103].

Importantly, the tumor contains products of nutrient metabolism that are toxic to the system, including excessive phosphate. Breaching toxin containment by destroying the tumor releases these intracellular toxins into the blood stream, causing phosphate toxicity in a life-threatening oncological emergency known as tumor lysis syndrome [104]. The effects of tumor lysis syndrome produce “acute kidney injury, uremia, and systemic end-organ damage, including renal failure and liver failure, potentially causing seizures, cardiac dysrhythmias, and death”. Notable electrolyte irregularities in tumor lysis syndrome include hyperphosphatemia and hypocalcemia, secondary to elevated serum phosphate.

Although considered a rare event, the mortality rate of tumor lysis syndrome for solid tumors is as high as 40% [105], demonstrating the potential importance of toxin containment in encapsulated tumors as a protective effect. Future studies need to investigate how changes in dietary phosphate overload affect encapsulation in tumorigenesis. The overall evidence reviewed in this paper suggests that increased expression of phosphate transporters sequesters excessive extracellular Pi during the promotion of tumorigenesis, and tumor encapsulation potentially prevents efflux of contained Pi ions from reentering the general circulation causing phosphate toxicity. A recent review demonstrated that a mouse model of tumor suppression produced cancer cachexia effects of sarcopenia, organ atrophy, bone disorders, and premature death, similar to effects of phosphate toxicity [106].

## 7. Future Studies

**Figure 4:**
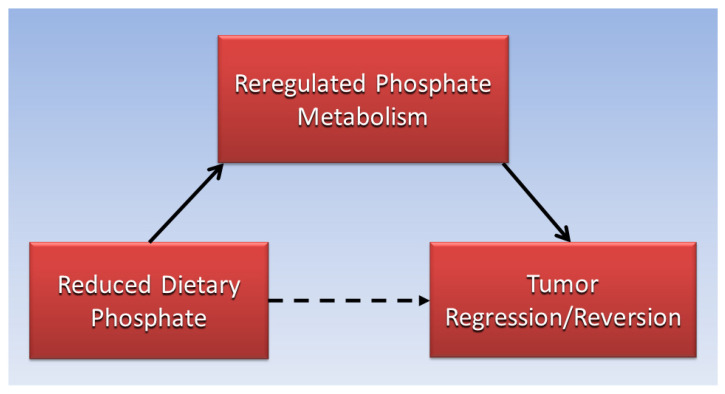
Proposed association of reduced dietary phosphate with tumor regression/reversion, mediated by reregulated phosphate metabolism.

The present paper proposes that reversing the transport and containment of an overload of Pi in tumors by reducing dietary phosphate intake has the potential to stimulate tumor regression and reversion. Figure 4 is a directed acyclic graph proposing that tumor regression and reversion is associated with reduced dietary phosphate through mediation by reregulated phosphate metabolism. Factors that potentially reduce dietary phosphate include sickness-associated anorexia [44], fasting-mimicking diets [52], and other diets low in phosphate such as certain types of ketogenic and plant-based diets. Phosphate binders also reduce the bioavailability of dietary phosphate absorbed in the intestines [107], and preclinical studies should investigate dietary phosphate intake levels associated with all of these dietary factors in tumor regression and reversion. Cancer reversion also occurs in malignant embryonic cells when transplanted into a healthy microenvironment [66], and studies should explore the effects of different concentrations of Pi in the microenvironment on malignant cell reversion. The uncontrolled activation of kinases is common in cancer [92], and studies are needed to prevent cancer initiation by restoring a proper balance of phosphorylated kinases and activated phosphatases using a reduced dietary phosphate load. Finally, autophagy is stimulated by the withdrawal of growth factors [58], and autophagy activated by the withdrawal of phosphate overload is a potential factor in tumor regression and reversion that warrants further study.

## 8. Conclusions

The spontaneous regression and reversion of tumors challenges the assumption that cancer is an irreversible genetic disease. Among the factors potentially responsible for tumor regression, the removal of a carcinogen by reducing elevated phosphate levels in the tumor microenvironment may be possible through reduced dietary phosphate intake. Tumor reversion depends on a healthy cellular microenvironment, which implies normal levels of cellular phosphate and a proper balance of kinases and phosphatases to reduce cell signaling in cancer. Fasting-mimicking diets and sickness-associated anorexia appear to stimulate the autophagy of tumors, and this effect may be due to a lower dietary phosphate intake. Encapsulated tumors have a protective effect potentially related to containment of toxic levels of phosphate. Preclinical studies of a low-phosphate diet for the regression and reversion of tumors in cancer patients are warranted.

## Figures and Tables

**Table 1:** Conclusions supporting tumor reversion [2].

“(a) Cancer cells display relevant plasticity, and their fate is not ‘irreversibly’ determined”.
“(b) It is possible to inhibit the phenotypic expression of the malignant characteristics of cancer cells mostly through epigenetic processes, although other mechanisms are likely to participate”. “(c) Depending on the tumor type and stage, some context-dependent conditions/constraints (such as those pertaining to the microenvironment of specific embryogenesis stages) can induce a phenotypic reversion of malignant cancer cells”. “(d) Gene mutations do not play a ‘causative’ role as the somatic mutation theory (SMT) posits, albeit they can be associated throughout the process of cancer development”.

**Table 2:** Nutrient metabolism factors in cancer [97] compared with dysregulated phosphate metabolism.

Nutrient Metabolism Factor in Cancer		Dysregulated Phosphate Metabolism
“(i) diet, the primary source of bodily nutrients which influences circulating metabolite levels”;		(i) dietary phosphate is the primary nutrient source of the circulating metabolite Pi;
“(ii) tissue of origin, which can influence the tumor’s reliance on specific nutrients to support cell metabolism and growth”;		(ii) phosphate influences most tissue types and stages of tumor growth;
“(iii) local microenvironment, which dictates the accessibility of nutrients to tumor cells”;		(iii) excessive extracellular Pi in local microenvironment is transported into tumor cells;
“(iv) tumor heterogeneity, which promotes metabolic plasticity and adaptation to nutrient demands”;		(iv) adaptation to oversupply of phosphate promotes tumorigenesis;
“(v) functional demand, which intensifies metabolic reprogramming to fuel the phenotypic changes required for invasion, growth, or survival”.		(v) dietary phosphate overload which may be reduced in tumor regression and reversion.

## Data Availability

No new data were created or analyzed in this study. Data sharing is not applicable to this article.

## Appendix A

**Table A1:** Phosphorus Dietary Reference Intakes (mg/day). Source: Institute of Medicine (2006) [108].

Age	EAR *	RDA *	AI *	UL *
Infants 0–6 months	—	—	100	—
Infants 7–12 months	—	—	275	—
Children 1–3 years	380	460	—	3000
Children 4–8 years	405	500	—	3000
Children 9–18 years	1055	1250	—	4000
Adults 19–70 years	580	700	—	4000
Adults over 70 years	580	700	—	3000
Pregnancy 18 years and under	1055	1250	—	3500
Pregnancy 19 years and over	580	700	—	3500
Lactation 18 years and under	1055	1250	—	4000
Lactation 19 years and over	580	700	—	4000

* AI = Adequate Intake. * EAR = Estimated Average Requirement. * RDA = Recommended Dietary Allowance. * UL = Tolerable Upper Intake Level (food, water, and supplements).

## Appendix B

**Table A2:** Foods high in phosphorus. Source: U.S. Department of Agriculture (USDA), FoodData Central. https://fdc.nal.usda.gov/, accessed on 29 May 2024.

Food (100 g)	Phosphorus (mg)
Leavening agents, baking powder, low-sodium	6870
Seeds, hemp seed, hulled	1650
Beverages, protein powder whey-based	1320
Gelatin desserts, dry mix, reduced calories, with aspartame	1290
Egg yoke, dried	1040
Fish, cod, Atlantic, dried and salted	950
Cheese spread, American or cheddar cheese base, reduced fat	931
Beverages, cocoa mix, no sugar added, powder	893
Wheat germ, crude	842
Cheese, Swiss, low fat	605
Biscuits, plain or buttermilk, dry mix	585
Wheat flout, white, all-purpose, self-rising, enriched	595
Beans, dry, light red kidney (0% moisture)	549
Pork, cured, bacon, cooked, broiled, pan-fried	533
Nuts, cashew nuts, raw	532
Flour, almond	512
Wheat, durum	508
Peanuts, Virginia, oil-roasted, with salt	506
Beef, variety meats and by-products, liver, cooked, braised	497
Cheese, provolone	496
Fish, sardine, Atlantic, canned in oil, drained solids with bone	490
Breast, cornbread, dry mix, enriched (includes corn muffin mix)	489
Cereals, QUAKER, instant oatmeal organic, regular	474
Cereals ready-to-eat, POST GRAPE-NUTS Cereal	467
Quinoa, uncooked	457
Cereals ready-to-eat, granola, homemade	431
Waffles, plain, frozen, ready-to-heat, toasted	429
Macaroni or noodles with cheese, made from reduced fat packaged mix, unprepared	403
Egg, yolk, raw, fresh	390
Lentils, dry	374
DENNY’S mozzarella cheese sticks	358
Fish, salmon, red, (sockeye), canned, smoked (Alaska Native)	350
Restaurant, Mexican, cheese quesadilla	347
Vegetarian meatloaf or patties	344
Pasta, whole-wheat, dry	343
Cheese, feta	337
Peanut butter, smooth style, without salt	335
Crackers, rye, wafers, plain	334
Beef, loin, tenderloin steak, boneless, separable lean only, trimmed to 0 fat, choice, cooked, grilled	321
Pork, fresh, loin, top loin (chops), boneless, separable lean only, with added solution, cooked, pan-broiled	320
School lunch, pizza, cheese topping, thin crust, whole grain, frozen, cooked	316
Chocolate, dark, 70–85% cacao solids	308
Ham, turkey, sliced, extra lean, prepackaged or deli	304
Bread, whole-wheat, commercially prepared, toasted	303
Chicken, broiler or fryers, giblets, cooked, simmered	289
Snacks, potato chips, white, restructured, baked	274
Doughnuts, cake-type, plain (included unsugared, old-fashioned)	260
Milk, canned, condensed, sweetened	253
Beef, ground, 85% lean meat/15% fat, crumbles, cooked, pan-browned	238
KFC, fried chicken, ORIGINAL RECIPE, thigh, meat and skin with breading	230
Rolls, dinner, whole-wheat	224
Bread, whole-wheat, commercially prepared	212
Nuts, macadamia nuts, raw	188
Cookies, sugar, refrigerated dough, baked	187
Bologna, beef, low fat	178
Turkey breast, low salt, prepackaged or deli, luncheon meat	162
Snacks, potato chips, lightly salted	148
Cake, chocolate, commercially prepared with chocolate frosting in-store bakery	137
Fast foods, potato, French fried in vegetable oil	125
Milk shakes, thick vanilla	115

## Appendix C

**Table A3:** Foods low in phosphorus. Source: USDA, FoodData Central. https://fdc.nal.usda.gov/, accessed on 29 May 2024.

Food (100 g)	Phosphorus (mg)
Oils: avocado; coconut; almond; olive; vegetable; soybean; peanut; canola; corn; sunflower; safflower; cottonseed; palm	0
Beverages, tea, herb, brewed, chamomile	0
Beverages, carbonated, ginger ale	0
Egg, white, raw, frozen, pasteurized	0
Beverages, coffee, brewed, prepared with tap water, decaffeinated	1
Salad dressing, French, cottonseed oil, home recipe	3
Soup, chicken broth or bouillon, dry, prepared with water	3
Beverages, coffee, instant, regular, prepared with water	3
Olives, ripe, canned	3
Alcoholic beverage, distilled, all (gin, rum, vodka, whiskey) 94 proof	4
Honey; sugars, brown: vinegar, distilled	4
Pineapple, raw	5
Blueberries, frozen, sweetened; apple juice, canned or bottled, unsweetened, without added ascorbic acid	7
Grapefruit, raw, white, all areas	8
Apples, red delicious, with skin, raw	9
Raw fruits: papaya; pears, bartlett	10
Syrups, table blends, pancake, with 2% maple, with added potassium	10
Melons, honeydew, raw	11
Milk, human, mature, fluid (for reference only)	14
Raw fruits: mangos; figs	14
Squash, winter, butternut, frozen, cooked, boiled, with salt	14
Melons, cantaloupe, raw	15
Alcoholic beverage, wine, light	15
Eggplant, cooked, boiled, drained, with salt	15
Beets, pickled, canned, solids and liquids	17
Orange juice, chilled, includes from concentrate, with added calcium and vitamins A, D, E	17
Cereals, CREAM OF WHEAT, instant, prepared with water, without salt	18
Beans, snap, green, canned, regular pack, solids and liquids	18
Vinegar, balsamic	19
Radishes, raw; Fast foods, coleslaw; sauerkraut, canned solids and liquids	20
Raw fruits: cherries, sweet: jackfruit: clementines	21
Raw fruits: bananas; peaches, yellow; blackberries; grapes, green, seedless	22
Nuts, coconut cream, canned, sweetened	22
Raw fruits: oranges, navels; apricots; strawberries	23
Raw vegetables: celery; cucumber with peel; tomatoes, red, ripe; peppers, sweet, yellow	24
Butter, whipped, with salt	24
Noodles, Japanese, soba, cooked	25
Prune juice, canned	25
Summer squash, zucchini, includes skin, frozen, cooked, boiled, drained, without salt	25
Onions, sweet, raw; collards, frozen, chopped, unprepared	27
Kale, frozen, unprepared	29
Mushrooms, shiitake, cooked, without salt	29
Carrots, cooked, boiled, drained, without salt	30
Lettuce, cos or romaine, raw	30
Cabbage, cooked, boiled, drained, without salt	33
Rice, white, steamed, Chinese restaurant	33
Sauce, pasta, spaghetti/marinara, ready-to-serve	34
Beans, snap, yellow, cooked, boiled, drained, with salt	39
Potatoes, boiled, cooked, without skin, flesh, with salt	40
Cauliflower, raw	44
Peas and carrots, frozen, cooked, boiled, drained, without salt	49
Spinach, raw	49
Broccoli, frozen, chopped, cooked, boiled, drained, with salt	49
Arugula, raw	52
Sweet potato, cooked, baked in skin, flesh, without salt	54
Avocados, raw, California	54
Brussels sprouts, cooked, boiled, drained, with salt	56
Dates, deglet noor, medjool	62
Figs, dried, uncooked	67
Candies, MARS SNACKFOOD US, 3 MUSKETEERS bar	69
DENNY’S hash browns	71
Sour cream, reduced fat	85
Yogurt, vanilla, non-fat	88
Corn, sweet, yellow, raw	89
Milk, producer, fluid, 3.7% milkfat	93
Milk, nonfat, fluid, with added vitamin A and vitamin D (fat free or skim)	107
Nuts, coconut meat, raw	113
